# Hypoxia-Inducible Factor-1α and Interleukin 33 Form a Regulatory Circuit to Perpetuate the Inflammation in Rheumatoid Arthritis

**DOI:** 10.1371/journal.pone.0072650

**Published:** 2013-08-15

**Authors:** Fanlei Hu, Lianjie Shi, Rong Mu, Jiaxin Zhu, Yingni Li, Xiaoxu Ma, Chun Li, Rulin Jia, Dongyue Yang, Yun Li, Zhanguo Li

**Affiliations:** Department of Rheumatology and Immunology, Peking University People’s Hospital, Beijing, China; Centro di Riferimento Oncologico, IRCCS National Cancer Institute, Italy

## Abstract

Hyperplasia of synovial fibroblasts, infiltration with inflammatory cytokines, and tissue hypoxia are the major characteristics of rheumatoid arthritis (RA). Interleukin 33 (IL-33) is a newly identified inflammatory cytokine exacerbating the disease severity of RA. Hypoxia-inducible factor-1α (HIF-1α) showed increased expression in RA synovium and could regulate a number of inflammatory cytokine productions. Nevertheless, its correlation with IL-33 remains largely unknown. Here, we showed that elevated levels of IL-33 were demonstrated in RA patient synovial fluids, with upregulated expression of HIF-1α and IL-33 in the synovial fibroblasts. Knocking down HIF-1α compromised IL-33 expression in rheumatoid arthritis synovial fibroblasts (RASF), while enforcing HIF-1α expression in RASF substantially upregulated IL-33 levels. HIF-1α promoted the activation of the signalling pathways controlling IL-33 production, particularly the p38 and ERK pathways. Moreover, we showed for the first time that IL-33 in turn could induce more HIF-1α expression in RASF, thus forming a HIF-1α/IL-33 regulatory circuit that would perpetuate the inflammatory process in RA. Targeting this pathological pathway and HIF-1α may provide new therapeutic strategies for overcoming the persistent and chronic inflammatory disease.

## Introduction

Rheumatoid arthritis (RA) is a chronic systemic inflammatory disease characterized by hyperplasia of synovial fibroblasts and variable degrees of bone and cartilage erosion. It causes considerable financial impact due to the high degree of functional impairment it induces; up to 30% of RA patients become permanently work disabled within 3 years of diagnosis if they do not receive medical treatment [1]. The pathogenesis remains to be fully elucidated.

Elevated levels of proinflammatory cytokine production are a key feature of synovial inflammation [2]. IL-6, IL-8, TNF-α, and IL-17 play dominant pathological roles in RA. Therapeutic strategies targeting IL-6 and TNF-α dramatically decrease signs and symptoms of RA, benefiting many RA patients [3], [4]. IL-33 is a newly identified cytokine belonging to the IL-1 family which also includes IL-1α, IL-1β, and IL-18 [5]. It is proved to be a ligand for the orphan receptor ST2 [5], [6]. IL-33 mRNA is broadly expressed in many tissues but is restricted in cellular distribution to smooth muscle cells, epithelial cells, fibroblasts, keratinocytes, dendritic cells, and activated macrophages. It is mainly located in the nucleus but can also be secreted in an autocrine and paracrine fashion [7], [8]. The pathogenic role of IL-33 in RA has been extensively studied. Previous studies demonstrated that IL-33 could exacerbate collagen-induced arthritis (CIA), inducing more production of proinflammatory cytokines and anti-collagen antibodies [9]. Anti-ST2 antibody that blocks IL-33 signalling attenuated the severity of experimental arthritis [10]; ST2^−/−^ mice developed impaired CIA [9]. Our previous studies showed that the serum levels of IL-33 were elevated in RA patients, which is associated with autoantibody production [11]. Our unpublished data revealed that IL-33 transgenic mice developed CIA with more severity and higher incidence. Nevertheless, the factors that control IL-33 production in RA remain largely unknown.

Synovial tissue hypoxia of the inflamed joints is one of the most important characteristics of RA. The hypoxic nature of the RA synovium was first revealed in 1970 by measuring oxygen tension in samples of synovial fluids of patients with a Clark-type electrode [12]. This was further confirmed by other studies using nuclear magnetic resonance spectroscopy, pimonidazole, as well as video arthroscopy [13], [14], [15]. Hypoxia-inducible factor 1α (HIF-1α), the key transcriptional factor in the hypoxic response, shows upregulated expression in RA [16]. In addition to hypoxia, HIF-1α is also regulated by some inflammatory stimuli, although the molecular pathways haven’t been fully characterized. Both TNF-α and IL-1β have been shown to increase HIF-1α mRNA in RASF [17], [18]. No study has revealed the effect of IL-33 on HIF-1α expression. Hypoxia as well as HIF-1α was involved in many pathways, such as angiogenesis, cell migration, and cell survival [19], [20], [21]. Our previous study showed that hypoxia and hypoxia-inducible factor-1α provoked toll-like receptor signalling-induced inflammation in RA. HIF-1α overexpression enhanced RASF-mediated expansion of inflammatory T cells, inducing further proinflammatory IFN-γ and IL-17 production [22]. Ahn et al. reported that HIF-1α induced the expression of IL-6, IL-8, and MMP-3 in RASF [23]. Given the essential roles of IL-33 in the pathogenesis of RA, it is important to understand the potential impact of HIF-1α on IL-33 expression in RA.

In this study, we demonstrated that RA patients showed higher levels of IL-33 than OA patients in the synovial fluids. RASF expressed more IL-33 along with higher levels of HIF-1α than OASF. HIF-1α promoted the activation of the signalling pathways controlling IL-33 production. Knocking down HIF-1α compromised IL-33 expression in RASF, while enforcing HIF-1α expression in RASF upregulated IL-33 levels. Moreover, IL-33 in turn induced more HIF-1α expression in RASF, thus forming a HIF-1α/IL-33 self-amplification circuit that perpetuates the inflammation in RA. These findings further illustrated the pathogenic role of HIF-1α in RA, and suggested it might be served as a potential target for RA therapy.

## Materials and Methods

### Patients, tissue specimens, and ethics statement

Synovial tissue specimens used for culturing OASF (n = 4) and RASF (n = 7) were obtained from patients during total knee replacement surgery or arthroscopy. All patients fulfilled the American College of Rheumatology 2009 criteria for RA and 1995 criteria for OA, and provided their written consents to participate in this study. The study protocols, consent forms, and consent procedure were approved by the Institutional Medical Ethics Review Board of the Peking University People’s Hospital.

### Culture and identification of OASF and RASF

The synovial tissue specimens were minced into small pieces and incubated with 4 mg/ml type I collagenase (Sigma-Aldrich, Bornem, Belgium, Germany) in Dulbecco’s modified Eagle’s medium (DMEM) at 37°C for 2 h. The cells were collected by filtering the suspension through nylon mesh (70 µm), followed by extensive washing with PBS. Then the cells were cultured in complete high-glucose DMEM (Hyclone, Logan, UT, USA) supplemented with 10% FBS and 1% antibiotics in a humidified 5% CO_2_ incubator. OASF and RASF at passages 4–6 were used for the study, which were negative (99%) for CD14, CD11b, CD3, and CD19 as identified by flow cytometry analyses.

### RASF treatment

For HIF-1α silencing assay, HIF-1α-specific siRNA and the control scrambled siRNA (Santa Cruz, CA, USA) were transfected into RASF according to the manufacturer’s instructions, respectively. Briefly, 5×10^4^ RASF were planted in 6-well plates per well overnight, then were transfected with 4 µl siRNA mentioned above using Transfection Reagent (Santa Cruz). 24 h later, the cells were tested for gene knocking-down efficiency and were used for realtime PCR and ELISA assays.

For HIF-1α overexpression assay, 5×10^4^ RASF per well in 6-well plates were transfected with 2 µg HIF-1α plasmid or the control vector. The cells were stimulated with TNF-α plus IL-1β 24 h later and subjected to analyses.

For IL-33 silencing assay, synthesized IL-33-specific siRNA-1 (5′-UUACCAUCAAC ACCGUCACTT-3′), siRNA-2 (5′-UUUACACCUAUAAACACUCTT-3′), and the control scrambled siRNA (5′-ACGUGACACGUUCGGAGAATT-3′) were transfected into RASF, respectively as described above. 24 h later, the cells were tested for the gene knocking down efficiency. Accordingly, the cells were examined for HIF-1α expression by RT-PCR and realtime PCR.

For IL-33 stimulation assay, 5×10^4^ RASF per well in 6-well plates were stimulated with different doses (1 ng/ml, 10 ng/ml, and 100 ng/ml) of recombinant endotoxin-free human IL-33 for 24 h, or were stimulated with 100 ng/ml recombinant endotoxin-free IL-33 for different time (12 h, 24 h, and 48 h, respectively). Then the cells were harvested for detection of HIF-1α expression.

### Reverse transcription-polymerase chain reaction (RT-PCR) and realtime PCR analyses

Total RNA was extracted from cells using TRIzol reagent (Invitrogen, Carlsbad, CA, USA) and was treated with TURBO DNase (Ambion, Austin, TX, USA) to eliminate contamination of genomic DNA. Reverse transcription was performed with the RevertAid First Strand cDNA synthesis kit (Fermentas, Glen Burnie, MD, USA) according to the manufacturer’s instructions. The resulting cDNA was subjected to PCR and realtime PCR analyses.

PCR was performed to analyze the expression of HIF-1α and IL-33 in RASF using the following primers: HIF-1α, sense primer: 5′-TGGCCTTGTGAAAAAGGGT-3′, antisense primer: 5′-TTGATGGGTGAGGAATGGGT-3′; and IL-33, sense primer: 5′-GGTGTTACTGAGTTACTATGAA-3′, antisense primer: 5′-GGAGCTCCACAGAGT GTTCCTTG-3′. The PCR products were separated by gel electrophoresis on 1% agarose.

Two-step realtime PCR was also performed to quantify the expression of HIF-1α and IL-33 using SYBR Green Master Mix (Applied Biosystems, Foster City, CA, USA) according to the manufacturer’s instructions. The primers were the same as those used for PCR as described above. Gene expression was quantified relative to the expression of the housekeeping gene GAPDH, and normalized to control by standard 2^−△△CT^ calculation.

### Western blot analysis

Cells were harvested and lysised in radioimmunoprecipitation assay (RIPA) lysis buffer (Thermo Fisher Scientific Inc., Rockford, IL, USA), and incubated on ice for 30 minutes. The lysates were centrifuged at 16,000 rpm at 4°C for 30 minutes. The supernatants were collected for Western blot analysis.

Western blot analysis was performed using reduced protein samples that were separated by SDS-polyacrylamide gel electrophoresis (PAGE) and transferred onto nitrocellulose membranes (Amersham Pharmacia, Little Chalfont, UK). The membranes were blocked with Tris-buffered saline containing 0.1% Tween-20 and 5% nonfat milk or 5% bovine serum albumin (for detecting phosphoproteins) at room temperature for 2 hours, and were incubated with primary antibodies, such as anti-phospho-ERK mAb, anti-phospho-p38 mAb, anti-ERK mAb, anti-p38 mAb (all from Cell Signalling Technology, Danvers, MA, USA), and anti-actin mAb (Tianjin Sungene Biotech Co., Ltd) at 4°C overnight. The membranes were washed three times with Tris-buffered saline containing 0.1% Tween-20 for 10 minutes each, and incubated with HRP-conjugated secondary antibodies (Cell Signalling Technology) at room temperature in the dark for 1 hour. The signal was visualized by enhanced chemiluminescence reagents (Millipore, Billerica, MA, USA).

### ELISA assay

Commercially available ELISA kits used for measuring IL-33 and HIF-1α were from R & D Systems (Minneapolis, MN, USA). For measuring IL-33 levels in the synovial fluids of OA patients (n = 30) and RA patients (n = 50), 100 µl synovial fluids without dilution per well were used in the 96-well ELISA plates. For measuring IL-33 levels in RASF, 100 µg total cell lysates per well were used. For measuring HIF-1α levels in RASF, 100 µg nuclear cell lysates per well were used. The detection was performed according to the manufacturer’s instructions.

### Statistical analysis

SPSS 17.0 (SPSS, Chicago, Illinois, USA) was used for statistical analysis. Differences between various groups were evaluated by Wilcoxon signed-rank test or Student’s *t* test, and were considered statistically significant when *P* was <0.05.

## Results

### Elevated levels of IL-33 in RA patient synovial fluids accompanied with upregulated expression of IL-33 and HIF-1α in RASF

**Figure 1:**
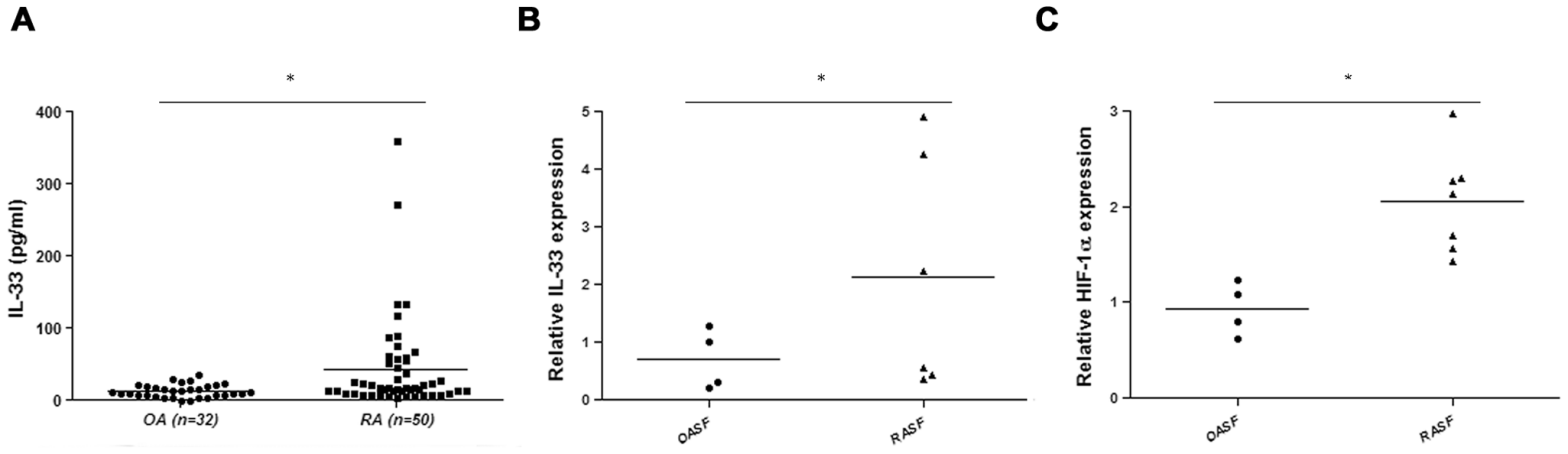
Elevated levels of IL-33 in RA patient synovial fluids. A, levels of IL-33 in the synovial fluids of RA patients (n = 50) and OA patients (n = 30) were detected by ELISA. The differences between the two groups were evaluated by Student’s *t* test. (*p<0.05). B, IL-33 expression levels were also determined by realtime PCR in the synovial fibroblasts of RA patients (RASF, n = 6) and OA patients (OASF, n = 4), one of the main producers of IL-33 (Wilcoxon signed-rank test, *p<0.05). C, Similarly, the expression levels of HIF-1α in RASF (n = 7) and OASF (n = 4) were also determined by realtime PCR (Wilcoxon signed-rank test, *p<0.05).

Much data has proved the significance of IL-33 in the pathogenesis of RA, so we first analyzed the levels of IL-33 in the synovial fluids of 50 cases of RA patient as well as 30 cases of age-matched OA patients by ELISA. A wide range of IL-33 levels were seen in RA patient synovial fluids, which were significantly higher than those in OA patient synovial fluids (Figure 1A). We then examined the expression of IL-33 in the synovial fibroblasts, one of the major IL-33 producing cells. Realtime PCR showed that RASF expressed higher levels of IL-33 than OASF (Figure 1B). Studies have revealed the correlation between HIF-1α and proinflammatory cytokine production in RA. Ahn et al. showed that HIF-1α could promote the expression of IL-8 in RASF [23], so we further detected HIF-1α expression in RASF. As expected, RASF revealed more HIF-1α expression than OASF, as demonstrated by realtime PCR analysis (Figure 1C).

### Effects of HIF-1α on IL-33 expression in RASF

**Figure 2:**
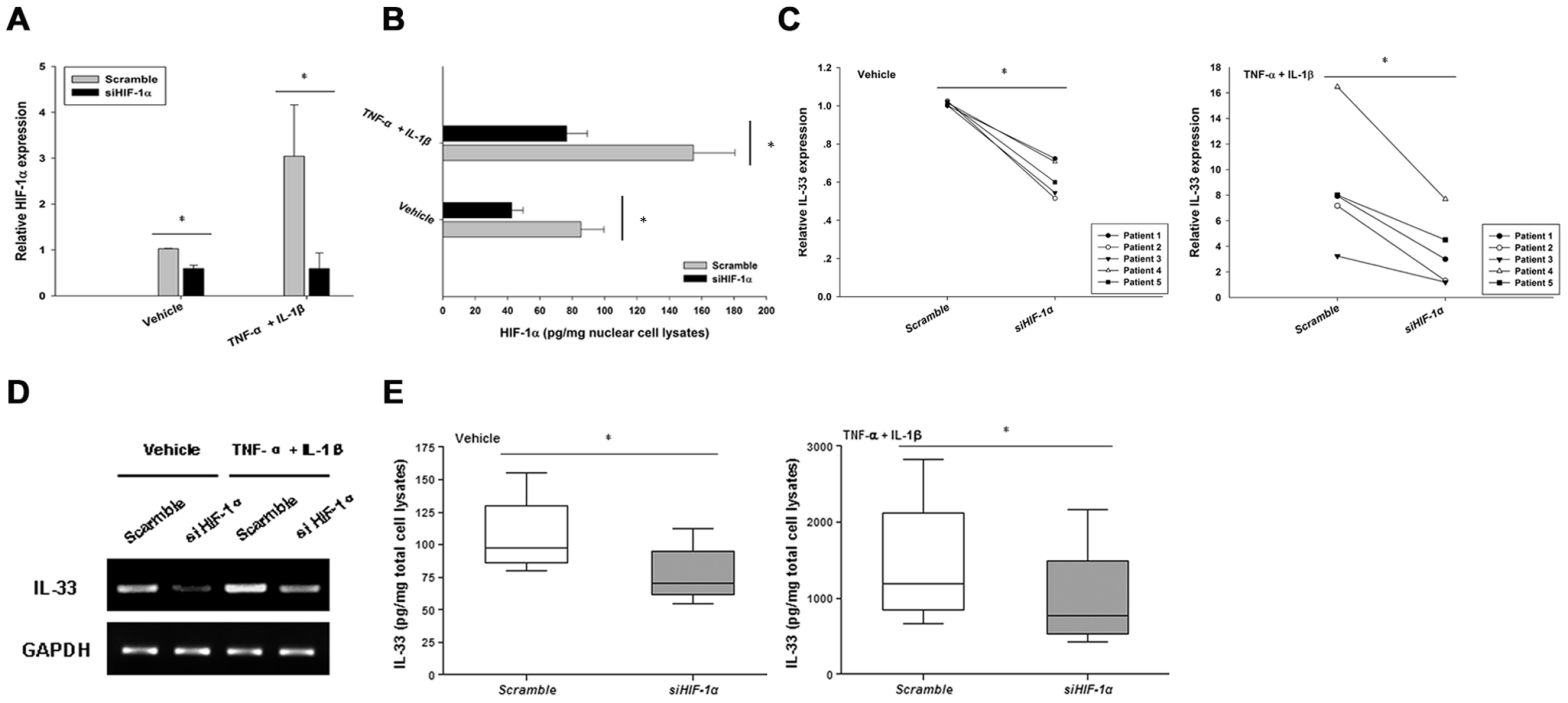
Knocking-down HIF-1α compromised IL-33 expression in RASF. A, RASF were transfected with HIF-1α siRNA or the control scramble siRNA. 24 h later, the cells were stimulated with 10 ng/ml TNF-α plus 2 ng/ml IL-1β. HIF-1α knockdown efficiency was confirmed by realtime PCR (A) and cell lysate ELISA (B) analyses. The IL-33 transcriptional levels were determined by realtime PCR (C) and RT-PCR (D) 4 h after stimulation. And also, the protein levels of IL-33 were assayed by cell lysate ELISA analysis (E) 24 h after stimulation (Wilcoxon signed-rank test, *p<0.05). The results were presented as mean±SEM of five independent experiments.

We next sought to determine the effects of HIF-1α on IL-33 expression. We first knocked down HIF-1α expression in RASF by specific siRNA, which was confirmed by realtime PCR (Figure 2A) and cell lysate ELISA (Figure 2B) analyses. Realtime PCR analysis showed that when HIF-1α was silenced, RASF demonstrated compromised IL-33 expression, both under the silenced and TNF-α- plus IL-1β-activated conditions (Figure 2C). And this was further confirmed by RT-PCR (Figure 2D) and ELISA (Figure 2E) analyses.

**Figure 3:**
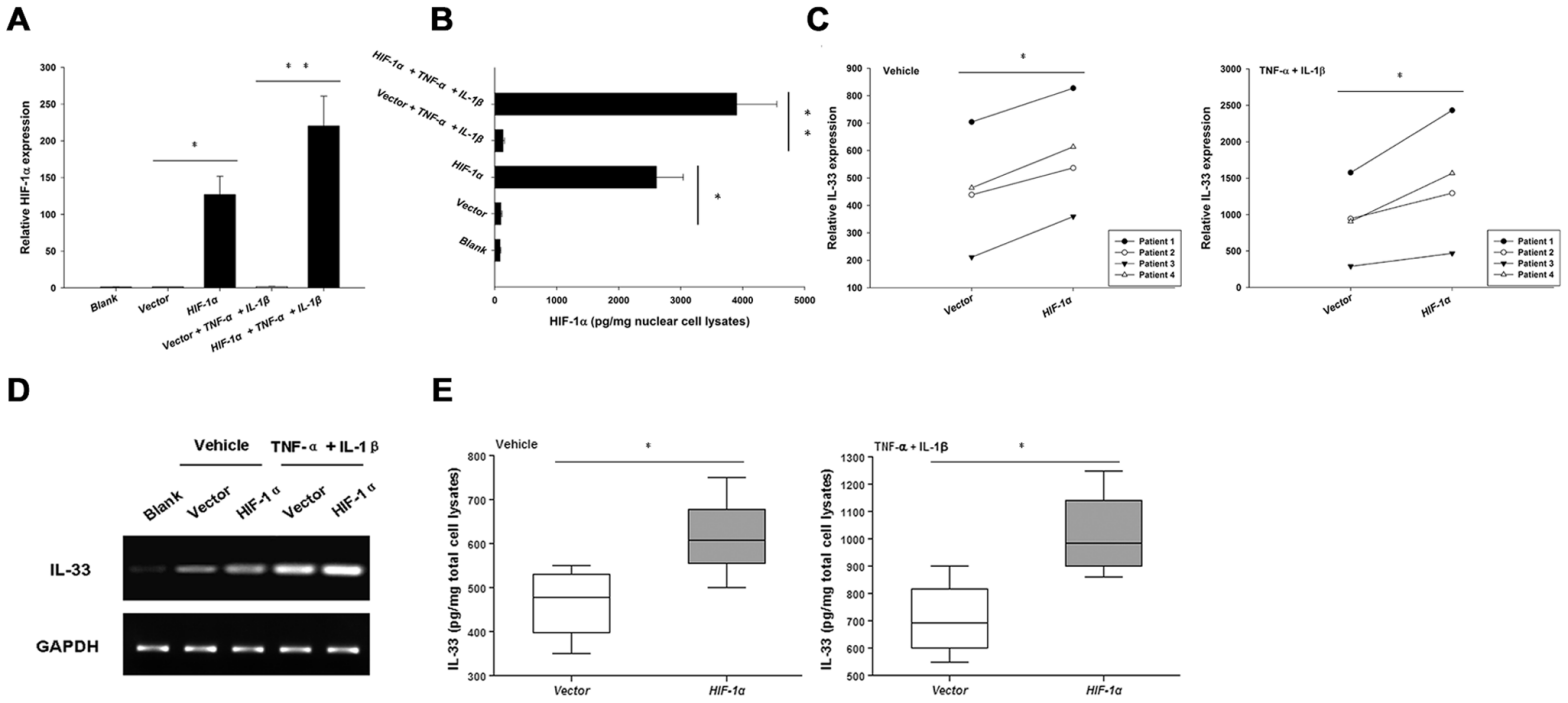
Enforced HIF-1α expression in RASF upregulated IL-33 levels. A, RASF were transfected with pcDNA3.1 or pcDNA3.1-HIF-1α for 24 h, then were stimulated with 10 ng/ml TNF-α plus 2 ng/ml IL-1β. The HIF-1α mRNA levels (A) as well as protein levels (B) were first assayed to confirm the overexpression. Cells were harvested 4 h after stimulation for realtime PCR (C) and RT-PCR (D) analyses of IL-33. 24 h later, cells were collected for measuring IL-33 protein levels (E) by cell lysate ELISA (Wilcoxon signed-rank test, *p<0.05, **p<0.001). The results were presented as mean±SEM of four independent experiments.

To further confirm the contribution of HIF-1α to IL-33 expression, we enforced the expression of HIF-1α in RASF by transfecting an HIF-1α plasmid, the presence of which was confirmed by realtime PCR (Figure 3A) and cell lysate ELISA (Figure 3B) analyses. HIF-1α overexpression significantly enhanced IL-33 expression in RASF, particularly under the TNF-α- plus IL-1β-activated conditions, as demonstrated by RT-PCR and realtime PCR analyses (Figure 3C, D). ELISA further validated this upregulated expression (Figure 3E).

### HIF-1α promoted activation of the signalling pathways controlling IL-33 expression

**Figure 4:**
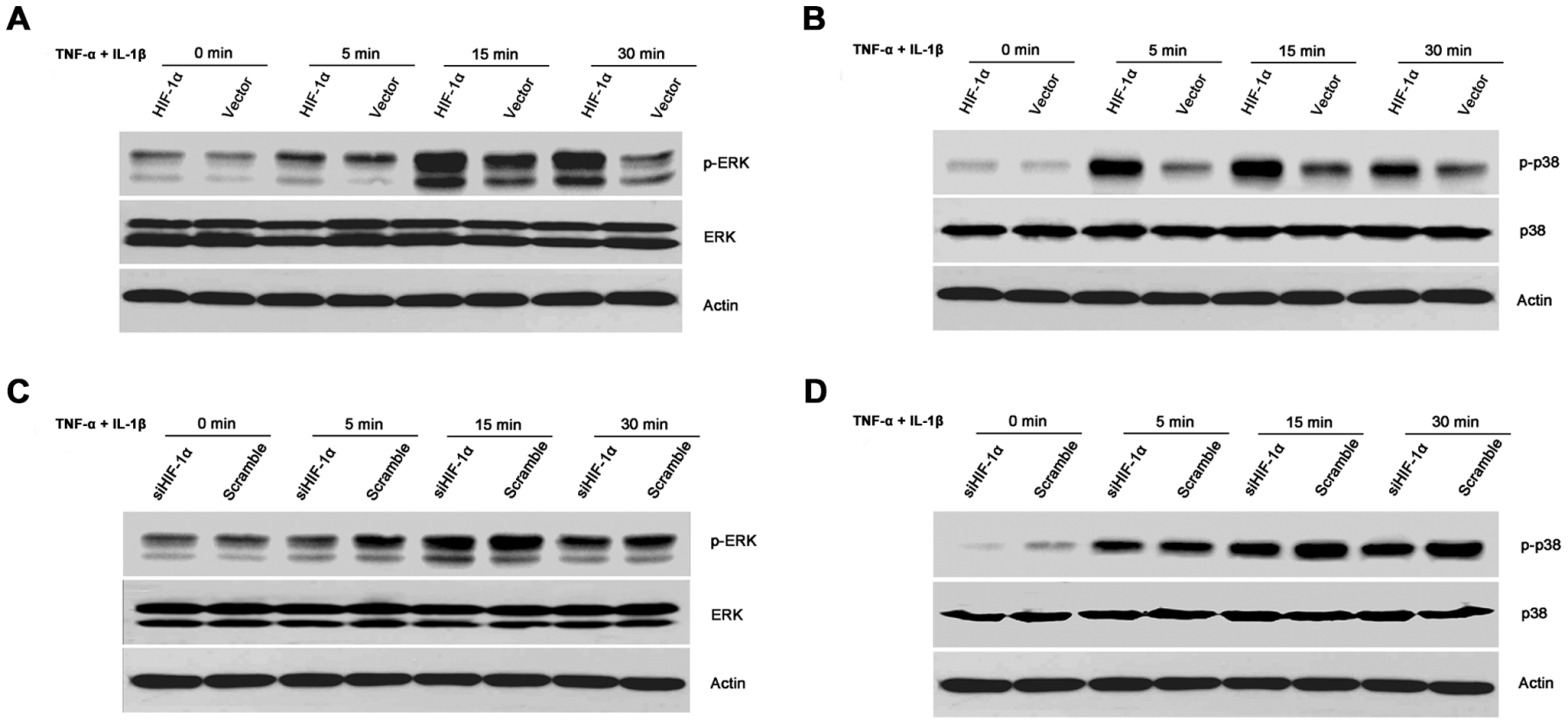
HIF-1α activated the signalling pathways controlling IL-33 expression. A-B, HIF-1α overexpression synergized with TNF-α and IL-1β to induce the activation of ERK and p38 pathways. RASF were transfected with pcDNA3.1 or pcDNA3.1-HIF-1α. 24 h later, the cells were stimulated with 10 ng/ml TNF-α plus 2 ng/ml IL-1β for 5 min, 15 min, and 30 min, respectively. Then the cells were lysised for assessment of the phosphorylation of ERK (A) and p38 (B) by Western blot. C-D, Silencing HIF-1α dampened TNF-α- plus IL-1β-induced ERK and p38 signalling pathway activation. RASF were transfected with HIF-1α siRNA or the control scramble siRNA for 24 h. Then the cells were stimulated with 10 ng/ml TNF-α plus 2 ng/ml IL-1β for 5 min, 15 min, and 30 min, respectively. After that, the cells were harvested for assaying the activation of the ERK (C) and p38 (D) pathways by Western blot. The results were representative of three independent experiments.

The p38 and ERK pathways played fundamental roles in TNF-α-induced production of IL-33 [24]. The potential involvement of HIF-1α in these pathways has also been studied in tenocytes [25]. Given the promoting effects of HIF-1α on IL-33 expression, we moved forward to assess the effects of HIF-1α on activating these two pathways. As shown in Figure 4A, and B, HIF-1α overexpression induced much stronger activation of the ERK pathway as well as the p38 pathway upon the stimulation of TNF-α plus IL-1β. And the effects could be seen from 5 minutes until 30 minutes after stimulation. Conversely, HIF-1α silencing by specific siRNA dampened TNF-α- plus IL-1β-induced ERK and p38 pathway activation (Figure 4C, D). All these suggest that HIF-1α played a fundamental role in activating the signalling pathways controlling IL-33 expression.

### IL-33 in turn induced more HIF-1α expression in RASF, forming a HIF-1α/IL-33 regulatory circuit

**Figure 5:**
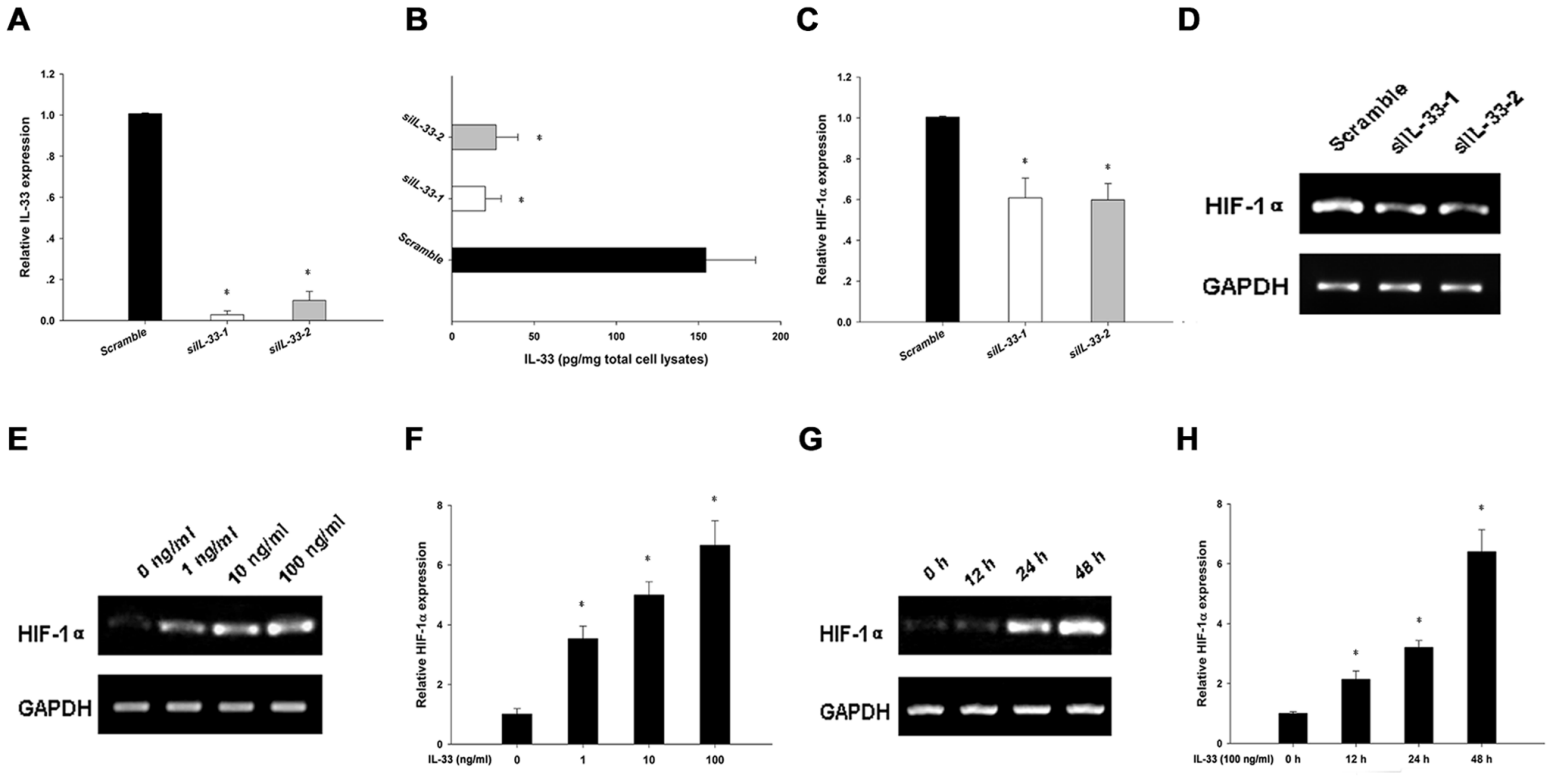
IL-33 enhanced HIF-1α expression in RASF, forming a self-amplification circuit. A-D, Knocking-down endogenous IL-33 compromised HIF-1α expression in RASF. RASF were transfected with IL-33 siRNAs or the control scramble siRNA. 24 h later, IL-33 knocking-down efficiency was determined by realtime PCR (A) and cell lysate ELISA (B). Accordingly, HIF-1α expression levels were assessed by realtime PCR (C) and RT-PCR (D) after IL-33 was silenced (Wilcoxon signed-rank test, *p<0.05 *vs.* the control scramble siRNA). E-H, Exogenous IL-33 stimulation promoted HIF-1α expression in RASF. RASF were stimulated with different doses (1 ng/ml, 10 ng/ml, and 100 ng/ml, respectively) of recombinant endotoxin-free human IL-33 for 24 h, then were assayed for HIF-1α expression by RT-PCR (E) and realtime PCR (F) analyses. Similarly, after stimulation with 100 ng/ml recombinant endotoxin-free human IL-33 for different time points (12 h, 24 h, and 48 h, respectively), RASF were harvested for detecting HIF-1α expression by RT-PCR (G) and realtime PCR (H) analyses (Wilcoxon signed-rank test, *p<0.05 *vs.* vehicle control). The results were presented as mean±SEM of four independent experiments.

**Figure 6:**
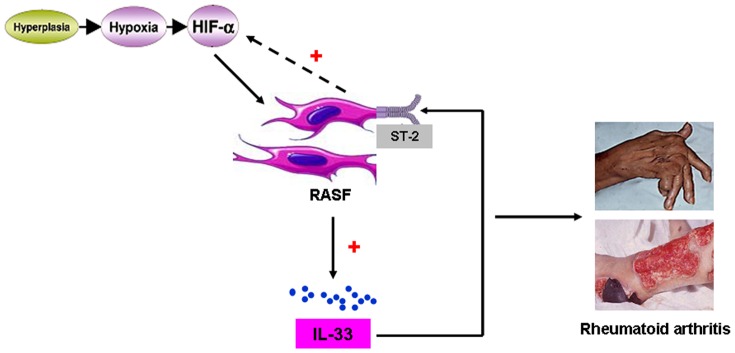
HIF-1α/IL-33 self-amplification circuit exacerbates the inflammation in RA. Rheumatoid arthritis synovial fibroblast (RASF) hyperplasia leads to tissue hypoxia, which induces upregulated expression of HIF-1α. This upregulated HIF-1α expression provokes IL-33 production by RASF, one of the main sources of IL-33. IL-33 in turn enhances HIF-1α expression in RASF, thus forming a self-amplification circuit that would perpetuate the inflammatory process in RA.

Studies have revealed that some inflammatory stimuli, particularly TNF-α and IL-1β could increase HIF-1α mRNA in RASF [17], [18]. So we further explored the role of IL-33 in inducing HIF-1α expression in RASF. We first knocked down IL-33 expression in RASF by specific siRNAs, as confirmed by realtime PCR (Figure 5A) and cell lysate ELISA (Figure 5B) analyses. RT-PCR and realtime PCR showed that this IL-33 silencing decreased HIF-1α mRNA levels in RASF (Figure 5C, D). We then stimulated RASF with recombinant endotoxin-free human IL-33. Our results showed that exogenous stimulation of IL-33 increased RASF HIF-1α expression levels both in a dose-dependent (Figure 5E, F) and in a time-dependent (Figure 5G, H) manner. This forms a HIF-1α/IL-33 regulatory circuit that would perpetuate the inflammatory process in RA (Figure 6).

## Discussion

In this study, we revealed that RA patients demonstrated higher levels of IL-33 than OA patients in the synovial fluids. Meanwhile, RASF expressed more IL-33 as well as HIF-1α than OASF. HIF-1α promoted the activation of the signalling pathways controlling IL-33 expression, particularly the p38 and ERK pathways, which eventually induced more IL-33 production in RASF. Moreover, IL-33 in turn stimulated HIF-1α expression in RASF, thus forming a HIF-1α/IL-33 self-amplification circuit that would exacerbate the inflammation in RA.

IL-33 is a cytokine with dual functions, acting both as a traditional cytokine through activation of the ST2L receptor complex and as an intracellular nuclear factor with transcriptional regulatory properties [26]. The amino terminus of the IL-33 molecule contains a nuclear localization signal and a homeodomain (helix-turn-helix-like motif) that can bind to heterochromatin as well as the H2A-H2B acidic pocket of nucleosomes in the nucleus [7], [27]. However, at present, the specific transcriptional targets of nuclear IL-33 are unclear. In this study, we demonstrated that IL-33, particularly the endogenous IL-33 could promote the transcriptional factor HIF-1α expression in RASF. This, at least in part, reveals one potential target for nuclear IL-33. Nevertheless, whether IL-33 could directly bind to the promoter of HIF-1α and facilitate its expression, remains to be further explored.

Several studies have revealed the pathogenic roles of IL-33 in RA. IL-33 was reported to be able to directly activate mast cells to secrete more proinflammatory cytokines and chemokines in the CIA model, including IL-1, IL-6, GM-CSF, MCP-1 and MIP-1, which in turn exacerbated the joint inflammation. In addition, these increased IL-1 and IL-6 could then promote Th17 cell development and function. Besides, IL-33 can also stimulate Th2 cells to produce more IL-5 and IL-13, which then enhanced B cell activation, leading to increased Ig production [9], [10]. In our previous study, we demonstrated that IL-33 serum concentration was significantly higher in patients with RA than in age-matched healthy controls, patients with primary Sjogren’s syndrome, and patients with osteoarthritis. Furthermore, the level of serum IL-33 decreased after anti-TNF-α treatment and was correlated with production of IgM and RA-related autoantibodies, including Rheumatoid Factor and anti-citrullinated protein antibodies [11]. Synovial fibroblasts are believed to be one of the main sources of IL-33 in RA, producing huge amounts of IL-33 in the presence of TNF-α and IL-1β stimulation [9]. Our unpublished data showed that IL-33 displayed key regulatory features of the biological activities of rheumatoid arthritis synovial fibroblasts. Knocking down IL-33 expression in RASF by specific siRNAs compromised their proliferation, production of proinflammatory cytokines, as well as invasion capacity. Recently, the role of IL-33 in bone metabolism and remodeling has also been studied. Mun and co-workers showed that IL-33 stimulated the formation of multi-nuclear osteoclasts from monocytes, and enhanced expression of osteoclast differentiation factors including TRAF6, nuclear factor of activated T cells cytoplasmic 1, c-Fos, c-Src, cathepsin K, and calcitonin receptor [28]. All these suggest that specific inhibition of IL-33 might provide a novel therapeutic approach for RA.

The microenvironmental conditions in the inflamed joint of RA patients are characterized by low partial oxygen pressure [12], [13], [14], [15]. HIF-1α, a key transcriptional factor in hypoxia, shows increased expression in RA [16]. In this study, we proved that HIF-1α primed IL-33 expression in RASF by activating the signalling pathways controlling IL-33 expression, particularly the p38 and ERK pathways. This explains, at least in part, the elevated levels of IL-33 in RA patient synovial fluids. The underlying mechanisms need to be further studied.

HIF-1α was involved in the pathogenesis of RA through many pathways. It promoted IL-8, MMPs, and VEGF expression in RASF, inducing cartilage destruction and angiogenesis [21], [23]. In addition, it could mediate the recruitment of monocytes, T and B lymphocytes in the rheumatoid synovium [19]. HIF-1α was also reported to directly enhance Th17 development via direct transcriptional activation of RORγt while attenuate Treg development via binding Foxp3 and targeting it for proteasomal degradation in mice [29], which might also contribute to RA pathogenesis in human. In our previous study, we showed that HIF-1α played a bridging role between the hypoxic response and the innate immune response. Hypoxia provoked toll-like receptor signalling-induced inflammation in RA through HIF-1α, especially TLR3 ligand polyIC-engaged inflammation. Moreover, overexpression of HIF-1α enhanced RASF-mediated expansion of inflammatory Th1 and Th17 cells, inducing a shift toward the proinflammatory state [22]. In this study, we further proved that HIF-1α and IL-33 formed a self-amplification circuit to exacerbate the inflammation in RA. This once again confirms the pathogenic role of HIF-1α in RA, suggesting it as a new therapeutic target.

In summary, we found for the first time that HIF-1α and IL-33 formed a regulatory circuit to perpetuate the inflammatory process in RA. Targeting this pathological pathway and HIF-1α may provide new therapeutic strategies for overcoming the persistent and chronic inflammatory disease.

## References

[1] Sokka T (2003) Work disability in early rheumatoid arthritis. Clin Exp Rheumatol 21: S71–74. 14969054

[2] Ulfgren AK, Lindblad S, Klareskog L, Andersson J, Andersson U (1995) Detection of cytokine producing cells in the synovial membrane from patients with rheumatoid arthritis. Ann Rheum Dis 54: 654–661. 7677442 10.1136/ard.54.8.654 PMC1009963

[3] van Oosterhout M, Levarht EW, Sont JK, Huizinga TW, Toes RE, et al (2005) Clinical efficacy of infliximab plus methotrexate in DMARD naive and DMARD refractory rheumatoid arthritis is associated with decreased synovial expression of TNF alpha and IL18 but not CXCL12. Ann Rheum Dis 64: 537–543. 15769913 10.1136/ard.2004.024927 PMC1755439

[4] Samson M, Audia S, Janikashvili N, Ciudad M, Trad M, et al.. (2012) Inhibition of IL-6 function corrects Th17/Treg imbalance in rheumatoid arthritis patients. Arthritis Rheum. 10.1002/art.34477 22488116

[5] Schmitz J, Owyang A, Oldham E, Song Y, Murphy E, et al (2005) IL-33, an interleukin-1-like cytokine that signals via the IL-1 receptor-related protein ST2 and induces T helper type 2-associated cytokines. Immunity 23: 479–490. 16286016 10.1016/j.immuni.2005.09.015

[6] Chackerian AA, Oldham ER, Murphy EE, Schmitz J, Pflanz S, et al (2007) IL-1 receptor accessory protein and ST2 comprise the IL-33 receptor complex. J Immunol 179: 2551–2555. 17675517 10.4049/jimmunol.179.4.2551

[7] Carriere V, Roussel L, Ortega N, Lacorre DA, Americh L, et al (2007) IL-33, the IL-1-like cytokine ligand for ST2 receptor, is a chromatin-associated nuclear factor in vivo. Proc Natl Acad Sci U S A 104: 282–287. 17185418 10.1073/pnas.0606854104 PMC1765450

[8] Luthi AU, Cullen SP, McNeela EA, Duriez PJ, Afonina IS, et al (2009) Suppression of interleukin-33 bioactivity through proteolysis by apoptotic caspases. Immunity 31: 84–98. 19559631 10.1016/j.immuni.2009.05.007

[9] Xu D, Jiang HR, Kewin P, Li Y, Mu R, et al (2008) IL-33 exacerbates antigen-induced arthritis by activating mast cells. Proc Natl Acad Sci U S A 105: 10913–10918. 18667700 10.1073/pnas.0801898105 PMC2491487

[10] Palmer G, Talabot-Ayer D, Lamacchia C, Toy D, Seemayer CA, et al (2009) Inhibition of interleukin-33 signaling attenuates the severity of experimental arthritis. Arthritis Rheum 60: 738–749. 19248109 10.1002/art.24305

[11] Mu R, Huang HQ, Li YH, Li C, Ye H, et al (2010) Elevated serum interleukin 33 is associated with autoantibody production in patients with rheumatoid arthritis. J Rheumatol 37: 2006–2013. 20682660 10.3899/jrheum.100184

[12] Lund-Olesen K (1970) Oxygen tension in synovial fluids. Arthritis Rheum 13: 769–776. 5495389 10.1002/art.1780130606

[13] Naughton DP, Haywood R, Blake DR, Edmonds S, Hawkes GE, et al (1993) A comparative evaluation of the metabolic profiles of normal and inflammatory knee-joint synovial fluids by high resolution proton NMR spectroscopy. FEBS Lett 332: 221–225. 7691662 10.1016/0014-5793(93)80636-9

[14] Peters CL, Morris CJ, Mapp PI, Blake DR, Lewis CE, et al (2004) The transcription factors hypoxia-inducible factor 1alpha and Ets-1 colocalize in the hypoxic synovium of inflamed joints in adjuvant-induced arthritis. Arthritis Rheum 50: 291–296. 14730627 10.1002/art.11473

[15] Ng CT, Biniecka M, Kennedy A, McCormick J, Fitzgerald O, et al (2010) Synovial tissue hypoxia and inflammation in vivo. Ann Rheum Dis 69: 1389–1395. 20439288 10.1136/ard.2009.119776 PMC2946116

[16] Hollander AP, Corke KP, Freemont AJ, Lewis CE (2001) Expression of hypoxia-inducible factor 1alpha by macrophages in the rheumatoid synovium: implications for targeting of therapeutic genes to the inflamed joint. Arthritis Rheum 44: 1540–1544. 11465705 10.1002/1529-0131(200107)44:7<1540::AID-ART277>3.0.CO;2-7

[17] Thornton RD, Lane P, Borghaei RC, Pease EA, Caro J, et al (2000) Interleukin 1 induces hypoxia-inducible factor 1 in human gingival and synovial fibroblasts. Biochem J 350 Pt 1: 307–312. PMC1221256 10926858

[18] Westra J, Brouwer E, Bos R, Posthumus MD, Doornbos-van der Meer B, et al (2007) Regulation of cytokine-induced HIF-1alpha expression in rheumatoid synovial fibroblasts. Ann N Y Acad Sci 1108: 340–348. 17893997 10.1196/annals.1422.035

[19] Ceradini DJ, Kulkarni AR, Callaghan MJ, Tepper OM, Bastidas N, et al (2004) Progenitor cell trafficking is regulated by hypoxic gradients through HIF-1 induction of SDF-1. Nat Med 10: 858–864. 15235597 10.1038/nm1075

[20] Biniecka M, Fox E, Gao W, Ng CT, Veale DJ, et al (2011) Hypoxia induces mitochondrial mutagenesis and dysfunction in inflammatory arthritis. Arthritis Rheum 63: 2172–2182. 21484771 10.1002/art.30395

[21] Hot A, Zrioual S, Lenief V, Miossec P (2012) IL-17 and tumour necrosis factor alpha combination induces a HIF-1alpha-dependent invasive phenotype in synoviocytes. Ann Rheum Dis 71: 1393–1401. 22532631 10.1136/annrheumdis-2011-200867

[22] Hu F, Mu R, Zhu J, Shi L, Li Y, et al.. (2013) Hypoxia and hypoxia-inducible factor-1alpha provoke toll-like receptor signalling-induced inflammation in rheumatoid arthritis. Ann Rheum Dis doi:10.1136/annrheumdis-2012–202444. 10.1136/annrheumdis-2012-202444 23644550

[23] Ahn JK, Koh EM, Cha HS, Lee YS, Kim J, et al (2008) Role of hypoxia-inducible factor-1alpha in hypoxia-induced expressions of IL-8, MMP-1 and MMP-3 in rheumatoid fibroblast-like synoviocytes. Rheumatology (Oxford) 47: 834–839. 18400834 10.1093/rheumatology/ken086

[24] Kunisch E, Chakilam S, Gandesiri M, Kinne RW (2012) IL-33 regulates TNF-alpha dependent effects in synovial fibroblasts. Int J Mol Med 29: 530–540. 22246057 10.3892/ijmm.2012.883 PMC3573710

[25] Millar NL, Reilly JH, Kerr SC, Campbell AL, Little KJ, et al (2012) Hypoxia: a critical regulator of early human tendinopathy. Ann Rheum Dis 71: 302–310. 21972243 10.1136/ard.2011.154229

[26] Haraldsen G, Balogh J, Pollheimer J, Sponheim J, Kuchler AM (2009) Interleukin-33 - cytokine of dual function or novel alarmin? Trends Immunol 30: 227–233. 19359217 10.1016/j.it.2009.03.003

[27] Roussel L, Erard M, Cayrol C, Girard JP (2008) Molecular mimicry between IL-33 and KSHV for attachment to chromatin through the H2A-H2B acidic pocket. EMBO Rep 9: 1006–1012. 18688256 10.1038/embor.2008.145 PMC2572127

[28] Mun SH, Ko NY, Kim HS, Kim JW, Kim do K, et al (2010) Interleukin-33 stimulates formation of functional osteoclasts from human CD14(+) monocytes. Cell Mol Life Sci 67: 3883–3892. 20532808 10.1007/s00018-010-0410-y PMC3399252

[29] Dang EV, Barbi J, Yang HY, Jinasena D, Yu H, et al (2011) Control of T(H)17/T(reg) balance by hypoxia-inducible factor 1. Cell 146: 772–784. 21871655 10.1016/j.cell.2011.07.033 PMC3387678

