# Correction: Hypoxia-Inducible Factor-1α and Interleukin 33 Form a Regulatory Circuit to Perpetuate the Inflammation in Rheumatoid Arthritis

**DOI:** 10.1371/annotation/f61a5d49-25e7-47ce-8509-6478df526886

**Published:** 2013-08-28

**Authors:** Fanlei Hu, Lianjie Shi, Rong Mu, Jiaxin Zhu, Yingni Li, Xiaoxu Ma, Chun Li, Rulin Jia, Dongyue Yang, Yun Li, Zhanguo Li

There was an error in the Funding statement. The correct version of the statement is:

This work was supported by grants from 973 program of China (2010CB529100)，and the Natural Science Foundation of China (81302554, 81030057, and 81072401), as well as by Peking University People’s Hospital Research and Development Funds (RDB2012-04). The funders had no role in study design, data collection and analysis, decision to publish, or preparation of the manuscript. 

